# Supplementary material for: Characterization of inositol lipid metabolism in gut-associated Bacteroidetes
Source: Nat Microbiol. 2022 Jun 20;7(7):986–1000. doi: 10.1038/s41564-022-01152-6 (PMC9246714; doi:10.1038/s41564-022-01152-6)
Supplement: Supplementary file 1 — Supplementary Results and Discussion, Figs. 1–5, Description of Supplementary Tables, and References. [file 41564_2022_1152_MOESM1_ESM.pdf]

nature portfolio

https://doi.org/10.1038/s41564-022-01152-6

Supplementary information

Characterization of inositol lipid metabolism in gut-associated Bacteroidetes

In the format provided by the authors and unedited

Supplementary Information for

Characterization of inositol lipid metabolism in gut-associated Bacteroidetes

Stacey L. Heaver [1], Henry H. Le [2], Peijun Tang [3], Arnaud Baslé [4], Claudia Mirretta Barone [1], Dai Long Vu [5], Jillian L. Waters [1], Jon Marles-Wright [4,6], Elizabeth L. Johnson [2], Dominic J. Campopiano [3], Ruth E. Ley [1,7]*

[1] Department of Microbiome Science, Max Planck Institute for Biology Tübingen, Tübingen 72076, Germany

[2] Division of Nutritional Sciences, Cornell University, Ithaca, NY 14853, USA

[3] School of Chemistry, University of Edinburgh, Edinburgh, Scotland, UK

[4] Newcastle University Biosciences Institute, Newcastle University, UK

[5] Mass Spectrometry Facility, Max Planck Institute for Biology Tübingen, Tübingen 72076, Germany

[6] School of Natural and Environmental Sciences, Newcastle University, UK

[7] Cluster of Excellence EXC 2124 Controlling Microbes to Fight Infections, Tübingen, Germany

* Corresponding author: Ruth E. Ley, ruth.ley@tuebingen.mpg.de

Supplementary Materials

Supplementary Results and Discussion 2

Supplementary Figures 5

Description of Supplementary Tables 11

Supplementary References 12

Supplementary Results and Discussion

Explanation of lipid structure determination

(In reference to Fig. 1 and Supplementary Figs. 1 + 2)

To analyze the chain length and branching structures of these lipids, we first performed fatty acyl methyl ester (FAME) analysis on total lipids extracted from WT BT, the iSPT strain at intervals from 0 to 100 ng/mL aTC induction of SPT, ΔBT_1522, and iSPTΔBT_1526 at 0 and 100 ng/mL aTC induction of SPT at n=3 per strain in rich (BHIS) medium (Supp. Fig. 1A). Induction of SL synthesis increased the proportional abundance of *anteiso*-C15 and *n*-16:0 fatty acids. Overall, *anteiso*-C15 was the most abundant fatty acid in WT BT, which conflicts with BT lipid structures portrayed recently in the literature as predominantly *iso*-branched (inferred by comparison to the *B. fragilis* lipid structures [1–3]). The identification of predominantly *anteiso*-C15 fatty acyl chains in BT, however, is consistent with the historical literature analyzing branching structure in various *Bacteroides* spp. [4].

Approach: To gain better resolution of the array of lipid structures in specific lipid classes, we separated total lipids by preparative TLC, and identified these lipid fractions by MALDI MS (one fraction including PI-DHC; another with PI-DAG and PS-DAG) (Supp. Fig. 1B). We performed FAME analysis on the PI-DAG/PS-DAG fraction to identify its branching structures (Supp. Fig. 1C). We excluded the FAME analysis on the PI-DHC fraction due to an inability to detect 3-OH lipids with our method and concerns over the efficiency of the *N*-methylation. Additionally, we collected LC-MS/MS data on PIP-DAG species with fingerprint fragments for acyl chain lengths.

Clarification of inositol glycerophospholipid structures: The lipid fraction including PI-DAG has PI-DAG 28:0 to PI-DAG 31:0 structures (Supp. Fig. 1B). Theoretically, the most likely acyl chain combinations producing these species would include: C14+C14 or C15+C13 (**PI-DAG 28:0**), C14+C15 or C13+C16 (**PI-DAG 29:0**), C15+C15 or C14+C16 (**PI-DAG 30:0**), C15+C16 or C14+C17 (**PI-DAG 31:0**). We collected LC-MS/MS data on the PIP-DAG species in the ΔBT_1525 strain (Supp. Fig. 2; Supp. Table 6), and identified that each of the three PIP-DAG parent masses

detected had at least one C15 chain: **PIP-DAG 29:0:** C14+C15; **PIP-DAG 30:0:** C15+C15; **PIP-DAG 31:0:** C15+C16. As PIP-DAG is the parent molecule of PI-DAG, and the FAME data of the PI-DAG fraction matches the LC-MS/MS chain length distribution, we thus characterized the predominant fatty acyl chain combinations for the following lipids as follows: **PI(P)-DAG 29:0:** *n*-14:0 + *anteiso*-C15; **PI(P)-DAG 30:0:** *anteiso*-C15 + *anteiso*-C15; **PI(P)-DAG 31:0:** *anteiso*-C15 + *n*-16:0. Given the abundance of PI-DAG 30:0 by MALDI-MS and PIP-DAG 30:0 by LC-MS/MS, we have represented PI(P)-DAG 30:0 in Fig. 1.

Clarification of inositol sphingolipid structures: The PI-DHC fraction has five lipid masses by MALDI MS (Supp. Fig. 1B) coinciding with PI-DHC 32:0 to PI-DHC 36:0. We were not able to detect the signature fragments indicating chain length in the MS/MS data for PI-DHC species. However, MS fragmentation data on BT DHC in the literature indicates that the sphinganine backbone of DHC generally stays consistent at a 17-carbon length, while the *N*-linked 3-OH fatty acyl chain varies [1]. A 17-carbon sphinganine backbone would result from a 15-carbon fatty acyl-CoA, for which the FAME data shows an abundance of *anteiso*-C15 (Supp. Fig. 1A). We could thus imagine the sphinganine backbone of a DHC is *anteiso*-branched. For the range of PI-DHC structures detected (32:0 to 36:0), assuming an *anteiso*-C17 sphinganine (which would not be detected in our FAME analysis), the *N*-linked fatty-acyl chains would be of 15-, 16-, 17-, 18-, and 19-carbon chain lengths. In the absence of more conclusive data on branching structure, we have chosen to represent the DHC species consistent with the branching of both acyl chains and C17 length of the sphinganine in existing literature [1] (for PI-DHC 34:0), but with *antesio*-, not *iso*-branching, to reflect *anteiso* abundance in our FAME results.

Structural and kinetic characterization of BT_1526 MIPS

(In reference to Fig. 3A-C, Ext. Data Fig. 4, Supp. Figs. 3-4)

The N-terminally His-tagged BT_1526 expressed well in a highly soluble form (~50 kDa in size, observed by SDS-PAGE and confirmed by electrospray ionization mass spectrometry, Supp. Fig. 3), and was purified to homogeneity by standard immobilized metal affinity chromatography methods. The colorimetric assay used to confirm the activity monitors the appearance of the inorganic phosphate released from the MIP product and not the G6P substrate [5] Kinetic analysis of BT_1526 MIPS operating on G6P are as follows: $K_m = 9.97 \pm 0.94$ mM, $V_{max} = 26.17 \pm 0.10$ μM/min, specific activity = 0.513 μmol/min/mg (Fig. 3B). This activity is in the range of the published specific activities of MIPS from *S. cerevisiae* (0.41 μmol/min/mg) [6,7], *Synechocystis* sp. (0.02 μmol/min/mg) [6], *A. fulgidus* (11.8 μmol/min/mg) [8], and *A. thaliana* (~0.1 μmol/min/mg) [9].

We further characterized the BT_1526 protein by determining the X -ray crystal structure to 2.0 Å resolution by molecular replacement using a model derived from the Archaeoglobus fulgidus MIPS structure (PDBID: 3QVT) (Fig. 3C; refinement statistics in Supp. Table 2). The overall 3D fold of BT_1526 is consistent with other members of this family, with a Rossman fold-like nucleotide binding domain (residues 4-244, 355-429) with an intercalated catalytic/dimerization domain (residues 245-354) [10–13]. The protein adopts a tetrameric dimer of dimers quaternary structure of approximately 188 kDa, with the dimerization domain forming an extended beta-sheet between monomers to create a saddle-like interface for the two dimers within the tetramer. Though the protein was purified without the addition of any cofactors or substrates, strong electron density consistent with the NAD+ cofactor was observed in the initial maps calculated after molecular replacement and the final structure contains an NAD+ molecule associated with each chain modeled at unit occupancy. Given the redox neutrality of the MIPS enzyme (i.e., it catalyzes substrate oxidation, then reduction) there is a clear benefit to the retention of the NAD+ in the active site for the lifetime of the protein, in agreement with NAD+ retention in MIPS from other species [14]. The catalytic active site region of the protein is well conserved among members of the MIPS family for which a structure has been determined, with a cluster of lysine and aspartic acid residues responsible for binding and orienting the G6P substrate for isomerization to the MIP product, highlighting the importance of these residues for the correct activity of the enzyme.

Supplementary Figures

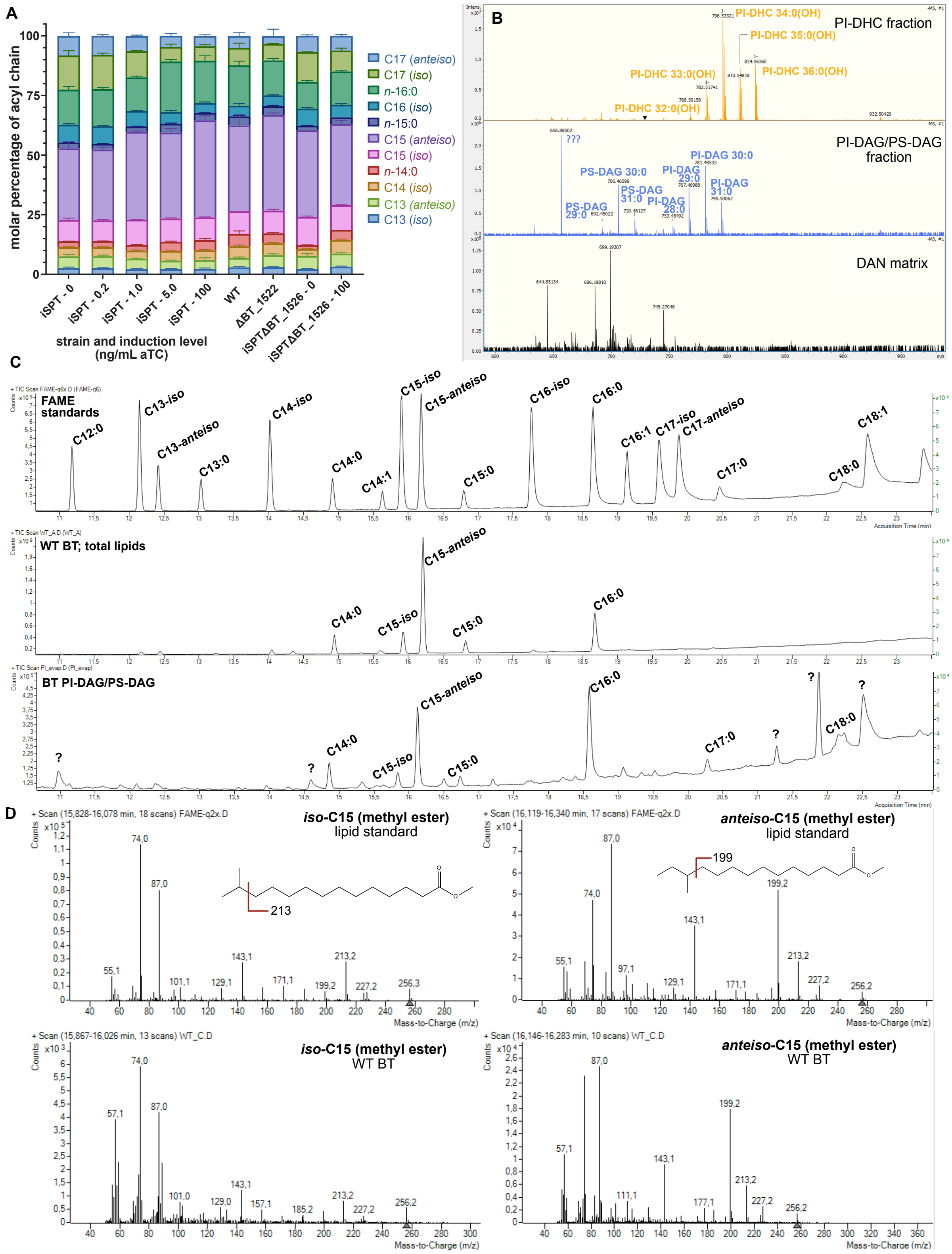

Supplementary Figure 1. Fatty acid methyl ester analysis of lipid chain length and branching in total and fractionated lipid extracts. (A) Molar proportion of non-hydroxylated fatty acyl abundance, measured by fatty acid methyl ester (FAME) analysis, in total lipids extracted from: WT BT; the iSPT strain at intervals of 0 to 100 ng/mL aTC induction of SPT; ΔBT_1522 and iSPTΔBT_1526 at 0 and 100 ng/mL aTC induction of SPT; n=3 per strain grown in rich (BHIS)

medium. Data are represented as mean values ± S.D. (B) MALDI MS data of lipid masses present in lipid fractions separated by preparative TLC. (C) Chromatograms of FAME GC-MS analysis of FAME standards, total lipids extracted from WT BT, and the PI-DAG/PS-DAG lipid fraction represented in panel B. (D) Ion spectra comparing *iso-* and *anteiso-*C15 methyl ester standards with methyl esters derived from WT BT lipids.

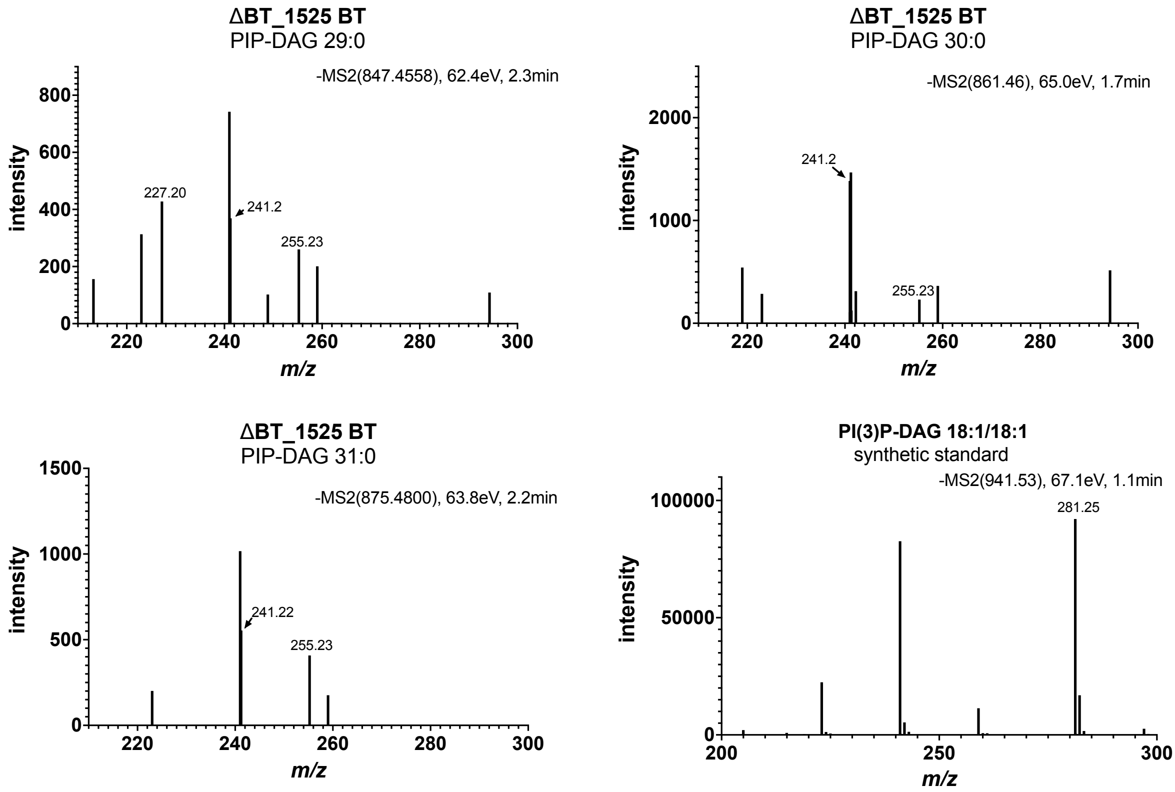

Supplementary Figure 2. MS2 spectra of PIP-DAG species in the ΔBT_1525 strain. The PIP-specific lipid extract of ΔBT_1525 was separated by LC-MS/MS, identifying ions indicative of fatty acyl fragments of specific chain length.

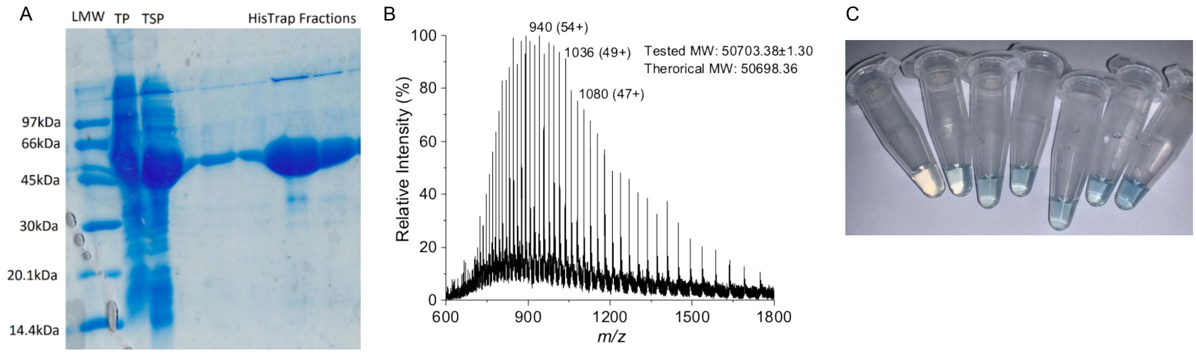

Supplementary Figure 3. Purification of BT_1526. (A) Expression and purification of recombinant BT_1526 MIPS purified from *E. coli*. The SDS-PAGE analysis shows the purity of the samples isolated by immobilized metal affinity chromatography (IMAC). The band between the 66k and 45 kDa corresponds to BT_1526 MIPS. (B) Electrospray ionization mass spectrometry (ESI-MS) analysis of the purified BT_1526 MIPS. Positive mode ion envelope with charge states annotated on particular masses. The predicted molecular weight matches well with the theoretical mass without the initial Met residue. (C) Typical color observed with the molybdenum blue assay of MIPS activity at 820 nm.

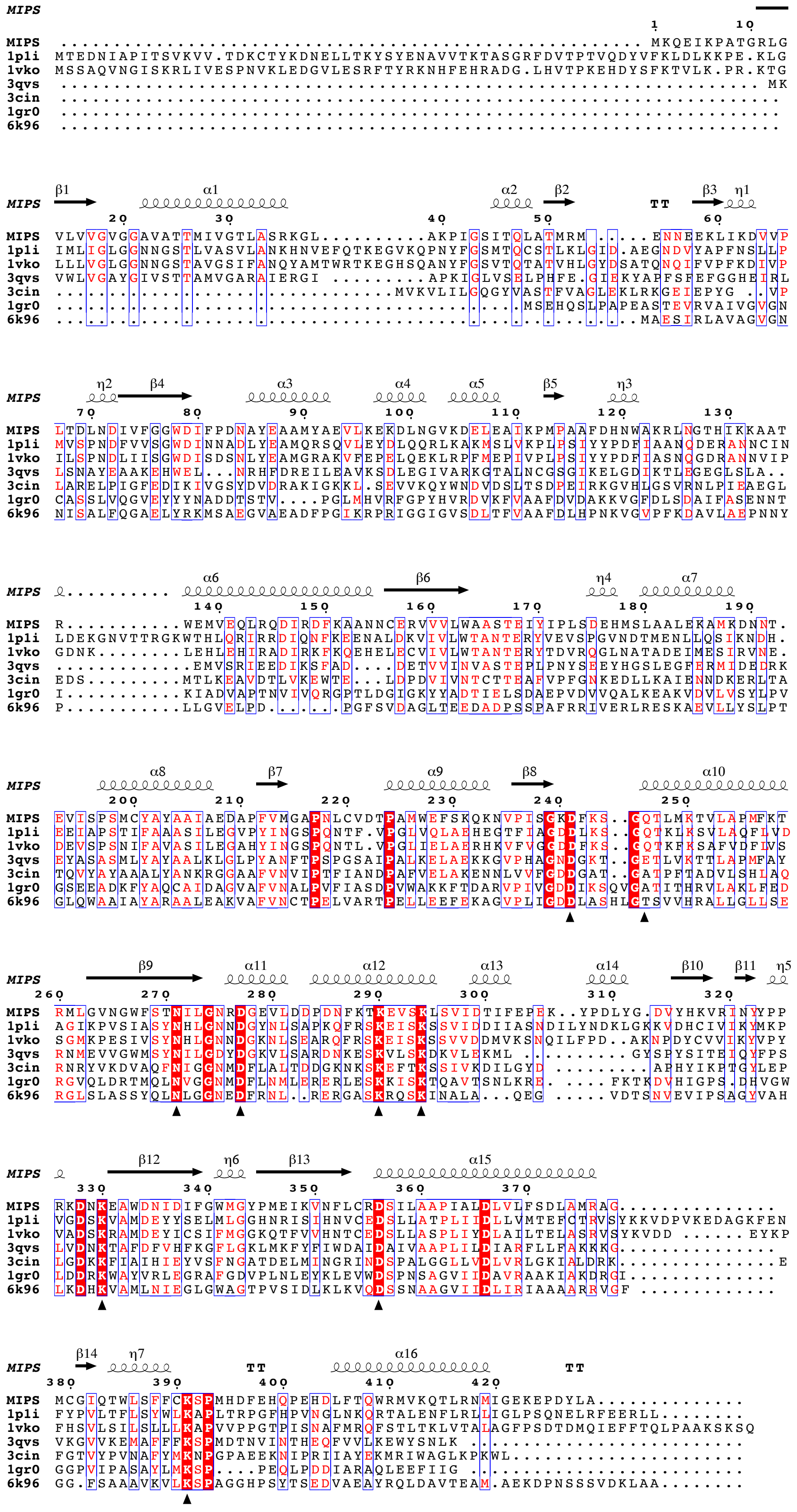

Supplementary Figure 4. MIPS structure and sequence comparison with active site residues. The sequences are: **MIPS (BT_1526), 1p1i** (*Saccharomyces cerevisiae*), **1vko** (*Caenorhabditis*

elegans), **3qvs** (*Archaeoglobus fulgidus*), **3cin** (*Thermotoga maritima* MSB8), **1gr0** (*Mycobacterium tuberculosis*) and **6k96** (*Streptomyces citricolor* Ari2). Secondary structural elements are annotated, active site residues are marked with an arrow and conserved residues marked with red shading (fully conserved) or pink shading (similar).

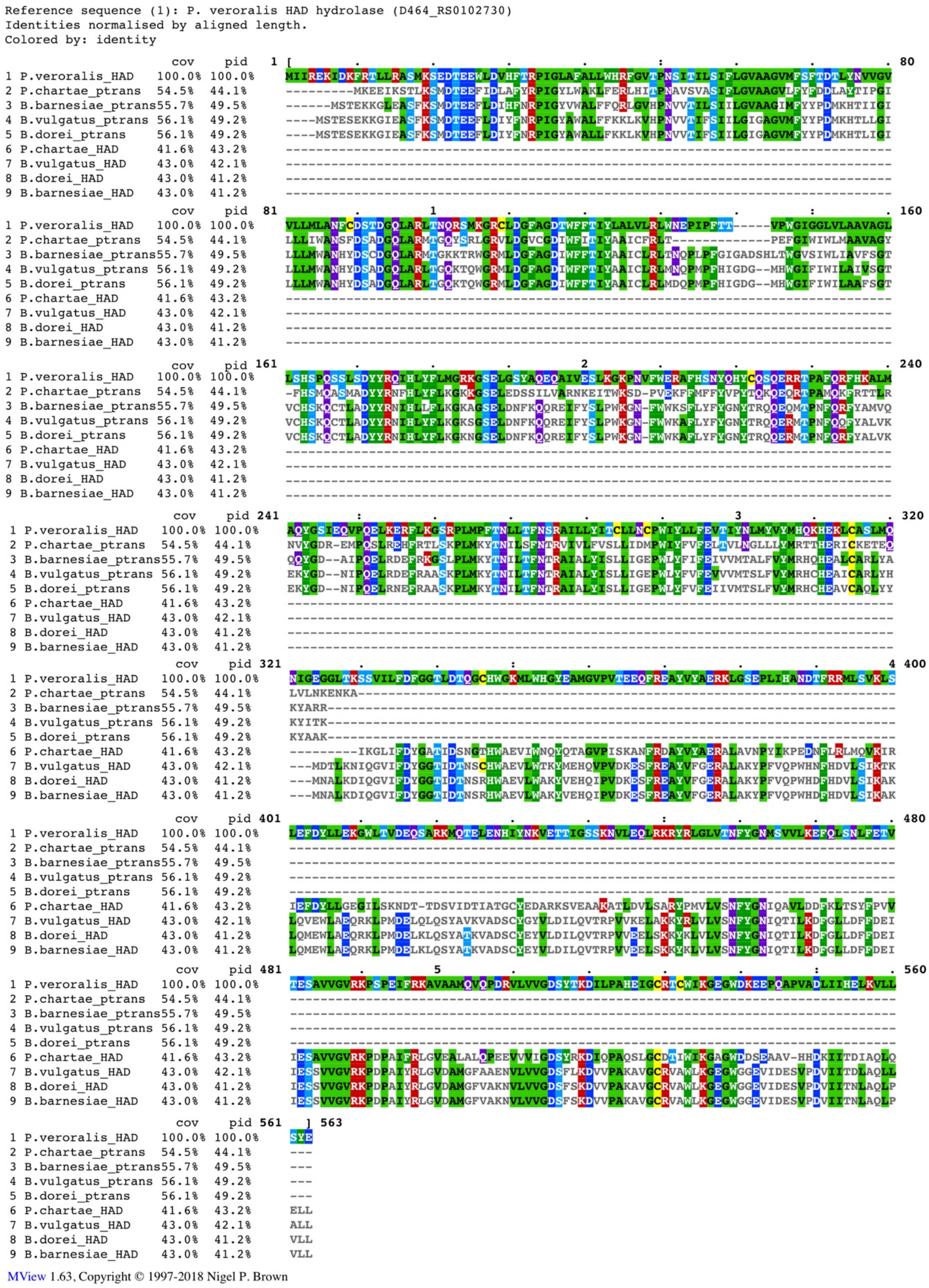

Supplementary Figure 5. Multiple sequence alignment of predicted proteins with putative involvement in an alternative inositol lipid metabolism cluster. Amino acid sequences from representative species containing the putative alternative inositol lipid metabolism cluster (*Parabacteroides chartae*, *Bacteroides barnesiae*, *Bacteroides vulgatus*, and *Bacteroides dorei*) HAD

hydrolase and CDP-alcohol phosphatidyltransferase, aligned to the *Prevotella veroralis* predicted fusion protein with homology to both of these proteins (D464_RS0102730). Alignment was performed using Clustal Omega with visualization by MView.

Description of Supplementary Tables

Supplementary Table 1: Strains and plasmids used in this study; primers used in the amplification of genomic regions prior to plasmid assembly via restriction digest or Gibson cloning; TetR cassette components, insertion locations, primers, and gene fragment for the generation of the inducible SPT BT strain. Components and assembly were inspired by Lim et al. 2017.

Supplementary Table 2: BT_1526 (MIPS) X-ray data collection and refinement statistics.

Supplementary Table 3: Top differentially expressed genes in the iSPT strain compared to the iSPTΔBT_1526 strain, both at 100 ng/mL aTC induction in minimal medium, with > 1.5 log2FC. Adjusted-P-value is Benjamini-Hochberg corrected. "CPS" column indicates the capsular polysaccharide synthesis (CPS) locus to which the gene belongs, when applicable.

Supplementary Table 4: Top differentially expressed genes in wild-type *B. theta* compared to the WTΔBT_1522 strain in minimal medium with > 1.5 log2FC. Adjusted-P-value is Benjamini-Hochberg corrected.

Supplementary Table 5: E-values of BLAST-P homology to the *B. thetaiotaomicron* inositol lipid cluster, or the putative alternative pathway (using *B. vulgatus* sequences: phosphatidyltransferase BVU_RS13105, HAD hydrolase BVU_RS13115, NTP transferase BVU_RS13095).

Supplementary Table 6: Theoretical and measured mass values for fatty acyl fragments shown in Supp. Fig. 2.

Supplementary References

1. Brown, E. M. *et al*. Bacteroides-Derived Sphingolipids Are Critical for Maintaining Intestinal Homeostasis and Symbiosis. *Cell Host Microbe* **25**, 668–680.e7 (2019).
2. Wieland Brown, L. C. *et al*. Production of α-galactosylceramide by a prominent member of the human gut microbiota. *PLoS Biol.* **11**, e1001610 (2013).
3. An, D. *et al*. Sphingolipids from a symbiotic microbe regulate homeostasis of host intestinal natural killer T cells. *Cell* **156**, 123–133 (2014).
4. Mayberry, W. R., Lambe, D. W., Jr & Ferguson, K. P. Identification of Bacteroides species by cellular fatty acid profiles. *Int. J. Syst. Evol. Microbiol.* **32**, 21–27 (1982).
5. Barnett, J. E., Brice, R. E. & Corina, D. L. A colorimetric determination of inositol monophosphates as an assay for D-glucose 6-phosphate-1L-myoinositol 1-phosphate cyclase. *Biochem. J* **119**, 183–186 (1970).
6. Chatterjee, A., Majee, M., Ghosh, S. & Majumder, A. L. sll1722, an unassigned open reading frame of Synechocystis PCC 6803, codes for L-myo-inositol 1-phosphate synthase. *Planta* **218**, 989–998 (2004).
7. Donahue, T. F. & Henry, S. A. myo-Inositol-1-phosphate synthase. Characteristics of the enzyme and identification of its structural gene in yeast. *J. Biol. Chem.* **256**, 7077–7085 (1981).
8. Chen, L., Zhou, C., Yang, H. & Roberts, M. F. Inositol-1-phosphate synthase from Archaeoglobus fulgidus is a class II aldolase. *Biochemistry* **39**, 12415–12423 (2000).
9. Donahue, J. L. *et al*. The Arabidopsis thaliana Myo-inositol 1-phosphate synthase1 gene is required for Myo-inositol synthesis and suppression of cell death. *Plant Cell* **22**, 888–903 (2010).
10. Norman, R. A. *et al*. Crystal structure of inositol 1-phosphate synthase from Mycobacterium tuberculosis, a key enzyme in phosphatidylinositol synthesis. *Structure* **10**, 393–402 (2002).
11. Jin, X. & Geiger, J. H. Structures of NAD+- and NADH-bound 1-l-myo-inositol 1-phosphate synthase. *Acta Crystallogr. D Biol. Crystallogr.* **59**, 1154–1164 (2003).
12. Stieglitz, K. A., Yang, H., Roberts, M. F. & Stec, B. Reaching for mechanistic consensus across life kingdoms: structure and insights into catalysis of the myo-inositol-1-phosphate synthase (mIPS) from Archaeoglobus fulgidus. *Biochemistry* **44**, 213–224 (2005).

13. Kudo, F., Tsunoda, T., Yamaguchi, K., Miyanaga, A. & Eguchi, T. Stereochemistry in the Reaction of the myo-Inositol Phosphate Synthase Ortholog Ari2 during Aristeromycin Biosynthesis. *Biochemistry* **58**, 5112–5116 (2019).
14. Chhetri, D. R., Adhikari, J. & Mukherjee, A. K. NAD+ mediated differential thermotolerance between chloroplastic and cytosolic L-myo-inositol-1-phosphate synthase from Diplopterygium glaucum (Thunb.) Nakai. *Prep. Biochem. Biotechnol.* **36**, 307–319 (2006).
