# Supplementary material for: Characterization of inositol lipid metabolism in gut-associated Bacteroidetes
Source: Nat Microbiol. 2022 Jun 20;7(7):986–1000. doi: 10.1038/s41564-022-01152-6 (PMC9246714; doi:10.1038/s41564-022-01152-6)
Supplement: Supplementary file 2 — Reporting Summary [file 41564_2022_1152_MOESM2_ESM.pdf]

nature research

Corresponding author(s): Ruth Ley

Last updated by author(s): 2022/04/12

Reporting Summary

Nature Research wishes to improve the reproducibility of the work that we publish. This form provides structure for consistency and transparency in reporting. For further information on Nature Research policies, see our Editorial Policies and the Editorial Policy Checklist.

Statistics

For all statistical analyses, confirm that the following items are present in the figure legend, table legend, main text, or Methods section.

n/a	Confirmed	
☐	☒	The exact sample size (n) for each experimental group/condition, given as a discrete number and unit of measurement
☐	☒	A statement on whether measurements were taken from distinct samples or whether the same sample was measured repeatedly
☐	☒	The statistical test(s) used AND whether they are one- or two-sided<br>*Only common tests should be described solely by name; describe more complex techniques in the Methods section.*
☒	☐	A description of all covariates tested
☐	☒	A description of any assumptions or corrections, such as tests of normality and adjustment for multiple comparisons
☐	☒	A full description of the statistical parameters including central tendency (e.g. means) or other basic estimates (e.g. regression coefficient) AND variation (e.g. standard deviation) or associated estimates of uncertainty (e.g. confidence intervals)
☐	☒	For null hypothesis testing, the test statistic (e.g. F, t, r) with confidence intervals, effect sizes, degrees of freedom and P value noted<br>*Give P values as exact values whenever suitable.*
☒	☐	For Bayesian analysis, information on the choice of priors and Markov chain Monte Carlo settings
☒	☐	For hierarchical and complex designs, identification of the appropriate level for tests and full reporting of outcomes
☒	☐	Estimates of effect sizes (e.g. Cohen's d, Pearson's r), indicating how they were calculated

Our web collection on statistics for biologists contains articles on many of the points above.

Software and code

Policy information about availability of computer code

Data collection	For mass spectrometry, Skyline version 21.1 and Compass Data Analysis version 4.3 were used.<br>NCBI Blast+ V2.11.0 was used for homology searches.
Data analysis	Mass spectrometry: Spectra were processed with MassLynx (V4.1).<br>RNA-seq: Quality assessment of reads was performed using FastQC (V0.11.8) pre- and post-quality filtering with bbduk (V38.90). Reads were aligned with bowtie2 (V2.3.5.1) and assigned using htseq-count (V0.11.2). Differential expression analysis was performed with EdgeR (V3.32.1) and limma (V3.46.0). Heatmaps were generated with pheatmap (V1.0.12).<br>Crystallography: The data set was integrated with XIA2 (V0.3.7.0) using DIALS (V3.9.1) and scaled with Aimless (V0.3.6). The space group was confirmed with Pointless (V1.10.20). The phase problem was solved by molecular replacement with Phaser (V2.7.17). The model was refined with refmac (V5.8) and manual model building with COOT (V0.1.2). The model was validated using COOT and Molprobity (V4.5). Figures were made with ChimeraX (V1.2).

For manuscripts utilizing custom algorithms or software that are central to the research but not yet described in published literature, software must be made available to editors and reviewers. We strongly encourage code deposition in a community repository (e.g. GitHub). See the Nature Research guidelines for submitting code & software for further information.

Data

Policy information about availability of data

All manuscripts must include a data availability statement. This statement should provide the following information, where applicable:

- Accession codes, unique identifiers, or web links for publicly available datasets
- A list of figures that have associated raw data
- A description of any restrictions on data availability

The BT_1526 MIPS structure analyzed during the current study is available in the Protein Data Bank repository, PDB ID:7NWR. RNA-seq reads and data are available at NCBI GEO (https://www.ncbi.nlm.nih.gov/geo/) under accession number GSE193734. Mass spectrometry files, and all unique strains generated in this study are available from the corresponding author upon request. All remaining data generated during this study are included in this published article and its supplementary information files.

Field-specific reporting

Please select the one below that is the best fit for your research. If you are not sure, read the appropriate sections before making your selection.

☒ Life sciences ☐ Behavioural & social sciences ☐ Ecological, evolutionary & environmental sciences

For a reference copy of the document with all sections, see nature.com/documents/nr-reporting-summary-flat.pdf

Life sciences study design

All studies must disclose on these points even when the disclosure is negative.

Sample size

The mouse experiment used n=8 mice per strain or competition pair tested, to minimize the number of mice used in a pilot test. We initially planned to use n=12 mice per group, in alignment with the mouse group size our lab has previously used for statistical testing of bacterial lipid-dependent physiological effects (1). We obtained however ethical approval for n=8 mice per group, as this was a pilot experiment, and this group size was sufficient to make our final conclusions. Capsule analysis was performed with n=3 capsule extractions per strain to compromise between cost of analysis and low overall abundance of the target of interest. On the basis of past studies showing significant results with the given sample size (2-4), RNA-seq analysis was performed with two biological replicates per strain, which we deemed to be sufficient due to low variability between replicates.

1. Johnson, E. L. et al. Sphingolipids produced by gut bacteria enter host metabolic pathways impacting ceramide levels. Nat. Commun. 11, 2471 (2020)
2. McNulty, N. P. et al. Effects of diet on resource utilization by a model human gut microbiota containing Bacteroides cellulosilyticus WH2, a symbiont with an extensive glycobiome. PLoS Biol. 11, e1001637 (2013)
3. Kijner, S., Cher, A. & Yassour, M. The Infant Gut Commensal Bacteroides dorei Presents a Generalized Transcriptional Response to Various Human Milk Oligosaccharides. Front. Cell. Infect. Microbiol. 12, 854122 (2022)
4. Dodd, D., Moon, Y.-H., Swaminathan, K., Mackie, R. I. & Cann, I. K. O. Transcriptomic Analyses of Xylan Degradation by Prevotella bryantii and Insights into Energy Acquisition by Xylanolytic Bacteroidetes. J. Biol. Chem. 285, 30261–30273 (2010)

Data exclusions

No data were excluded from the analyses.

Replication

With the exception of the mouse colonization, MIPS crystallization, electron microscopy, and RNA-seq, each experiment was performed a minimum of two times. Using the mouse samples, cecal colonization and in mouse-competition analyses were also performed twice. All attempts at replication were successful and supported the conclusions in the manuscript.

Randomization

For in vitro experiments, following strain generation, identical treatments were performed (e.g., lipid extraction and analysis, capsule extraction and analysis, AMP resistance and growth curves) so randomized allocation was not necessary, as a physical value was measured which is not influenced by the observer. Growth phase, temperature, and medium for in vitro bacterial growth were controlled and all conditions of a given experiment were performed in parallel. Electron microscopy images chosen are representative of the cell population of that strain. For mouse experiments, mice were randomly assigned to a treatment condition to randomize age and litter of origin.

Blinding

The investigators were not blinded during data collection. In the mouse experiment, the same investigators inoculated the mice and collected and analyzed downstream samples, preventing blinding. During computational analysis (RNA-seq), all data were subjected to the same analysis pipelines regardless of condition. During in vitro work with the strains, blinding was not used as the work was performed by a single investigator.

Reporting for specific materials, systems and methods

We require information from authors about some types of materials, experimental systems and methods used in many studies. Here, indicate whether each material, system or method listed is relevant to your study. If you are not sure if a list item applies to your research, read the appropriate section before selecting a response.

Materials & experimental systems

n/a	Involved in the study
☒	☐ Antibodies
☒	☐ Eukaryotic cell lines
☒	☐ Palaeontology and archaeology
☐	☒ Animals and other organisms
☒	☐ Human research participants
☒	☐ Clinical data
☒	☐ Dual use research of concern

Methods

n/a	Involved in the study
☒	☐ ChIP-seq
☒	☐ Flow cytometry
☒	☐ MRI-based neuroimaging

Animals and other organisms

Policy information about studies involving animals; ARRIVE guidelines recommended for reporting animal research

Laboratory animals	Female germ-free C57BL/6 mice aged 4-6 weeks, housed with a 12 hour light:dark cycle at 22±2 deg. C and ~55±10% humidity
Wild animals	The study did not involve wild animals.
Field-collected samples	The study did not utilize field-collected samples.
Ethics oversight	The mouse experiment was performed in accordance with the German legislation on protection of animals with permission to conduct the study obtained from the regional animal welfare committee of the Eberhard Karls Universität Tübingen, registration number EB 03/21 M.

Note that full information on the approval of the study protocol must also be provided in the manuscript.
