# Supplementary figures and images for: Characterization of inositol lipid metabolism in gut-associated Bacteroidetes
Source: Nat Microbiol. 2022 Jun 20;7(7):986–1000. doi: 10.1038/s41564-022-01152-6 (PMC9246714; doi:10.1038/s41564-022-01152-6)

Source Data

Fig. 2A

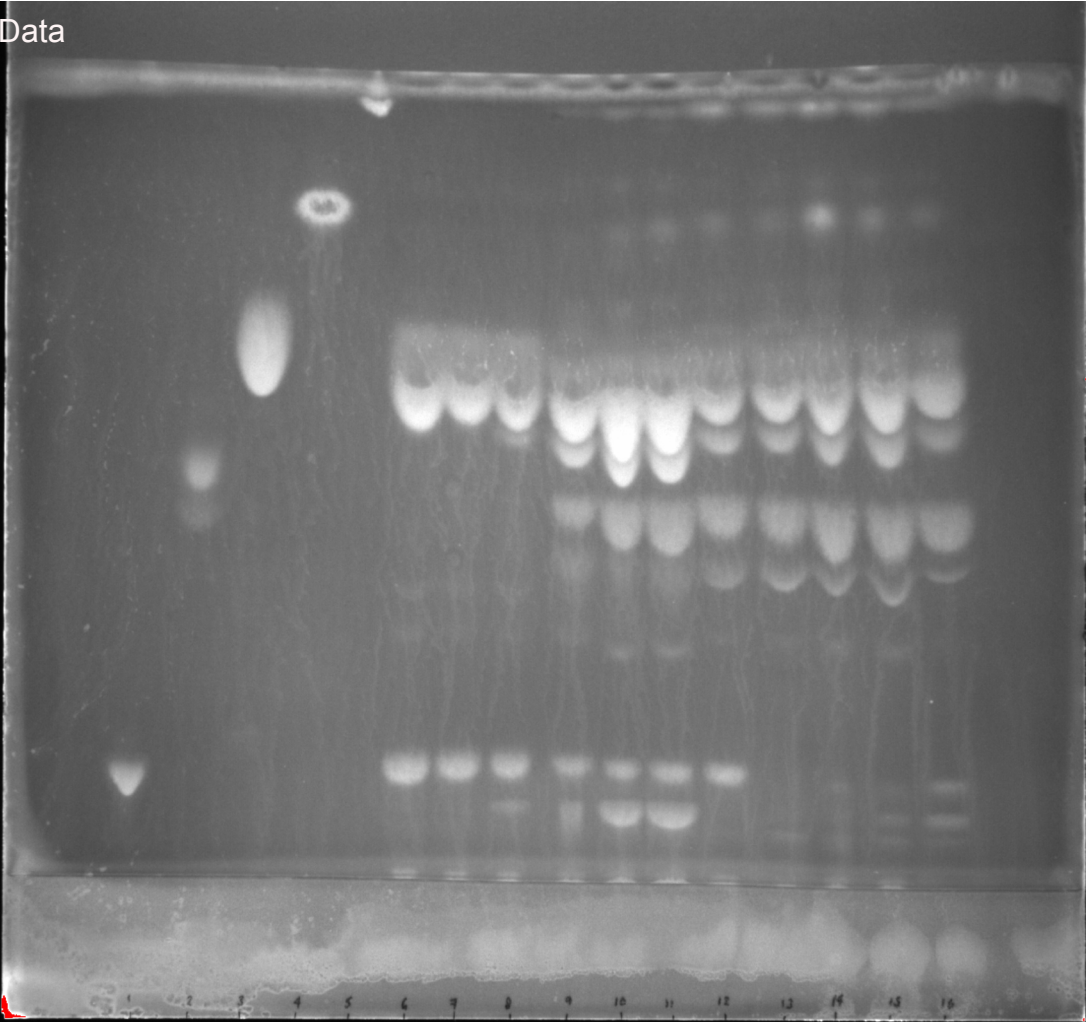

Source Data

Fig. 2B

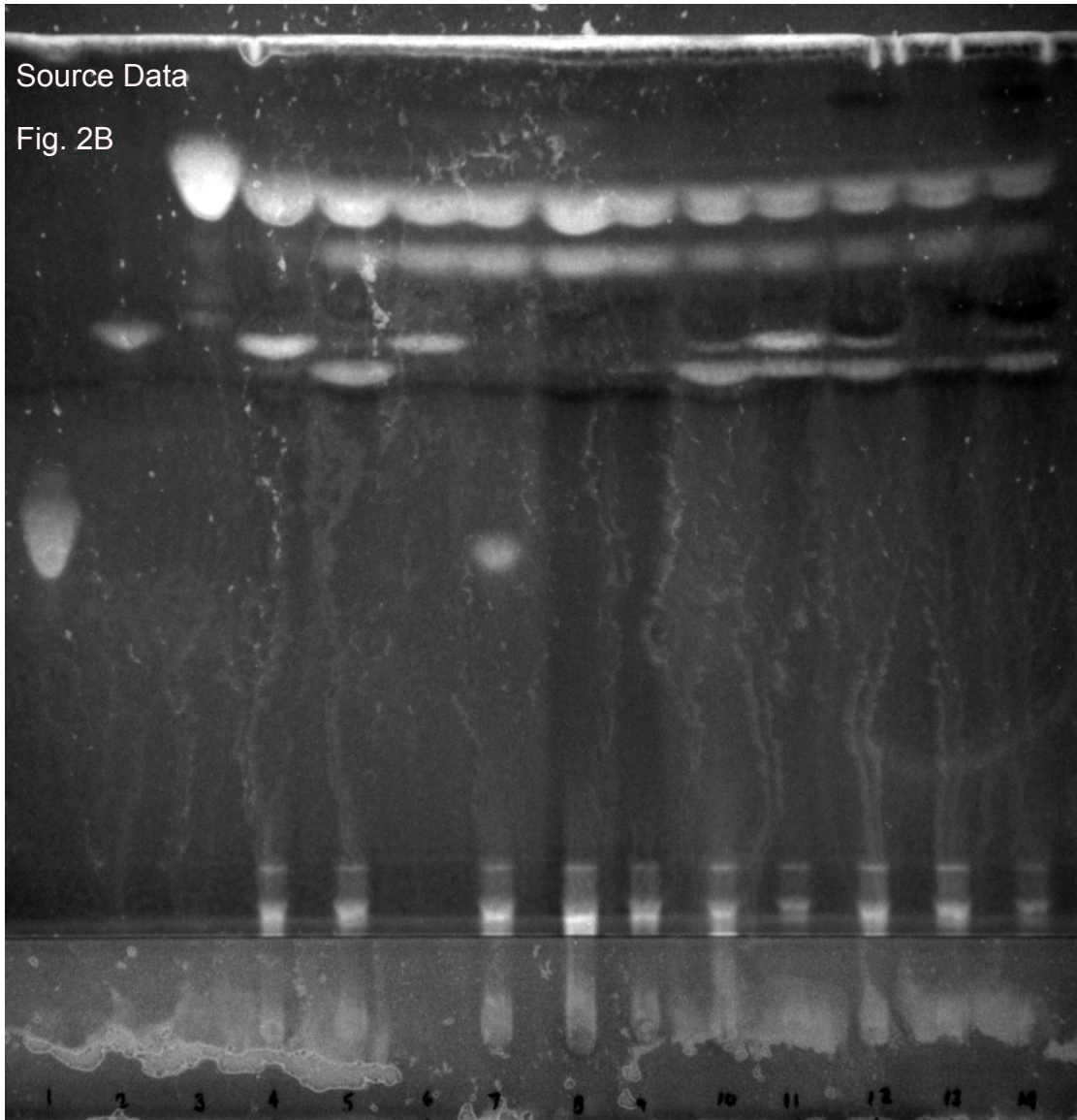

Supplement: Source Data Fig. 2 — Unprocessed TLC images. [file 41564_2022_1152_MOESM5_ESM.pdf]

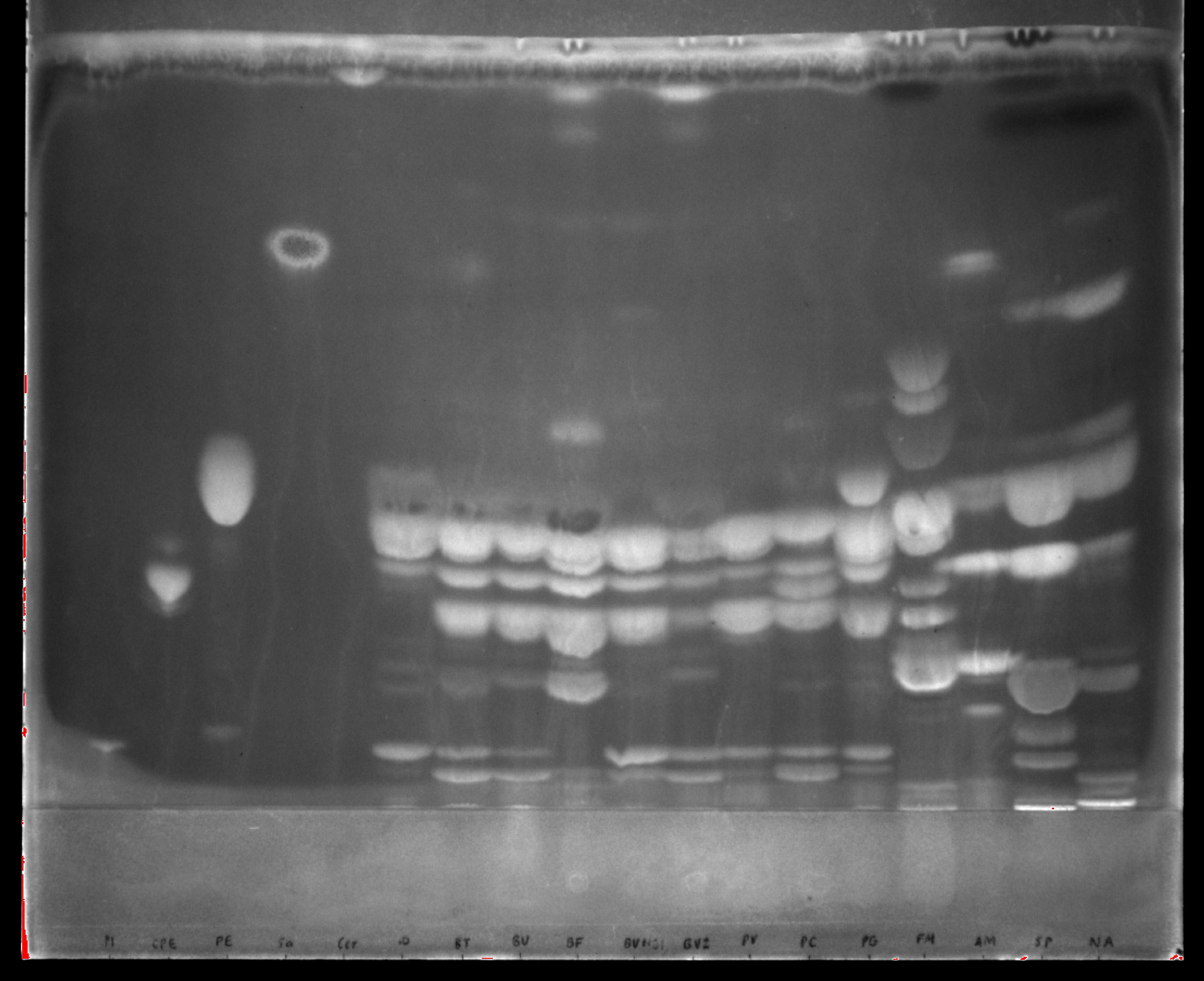

Supplement: Source Data Fig. 5 — Unprocessed TLC image. [file 41564_2022_1152_MOESM8_ESM.jpg]

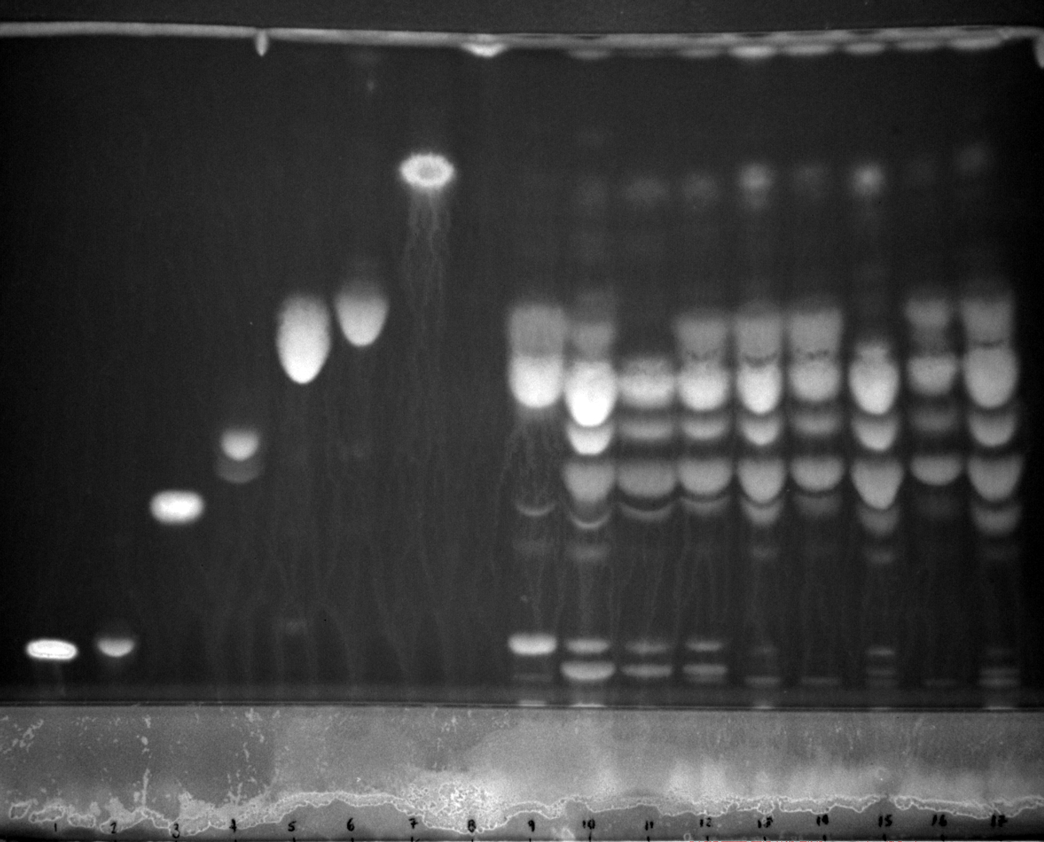

Supplement: Source Data Extended Data Fig. 3 — Unprocessed TLC image. [file 41564_2022_1152_MOESM9_ESM.jpg]
